# Dissonance-Based Eating Disorder Prevention Program Reduces Reward Region Response to Thin Models; How Actions Shape Valuation

**DOI:** 10.1371/journal.pone.0144530

**Published:** 2015-12-07

**Authors:** Eric Stice, Sonja Yokum, Allison Waters

**Affiliations:** 1 Oregon Research Institute, Eugene, United States of America; 2 University of Oregon, Eugene, United States of America; Leibniz Institute for Neurobiology, GERMANY

## Abstract

Research supports the effectiveness of a dissonance-based eating disorder prevention program wherein high-risk young women with body dissatisfaction critique the thin ideal, which reduces pursuit of this ideal, and the theory that dissonance induction contributes to these effects. Based on evidence that dissonance produces attitudinal change by altering neural representation of valuation, we tested whether completing the *Body Project* would reduce response of brain regions implicated in reward valuation to thin models. Young women with body dissatisfaction were randomized to this intervention or an educational control condition, completing assessments and fMRI scans while viewing images of thin versus average-weight female models at pre and post. Whole brain analyses indicated that, compared to controls, *Body Project* participants showed greater reductions in caudate response to images of thin versus average-weight models, though participants in the two conditions showed pretest differences in responsivity of other brain regions that might have contributed to this effect. Greater pre-post reductions in caudate and putamen response to thin models correlated with greater reductions in body dissatisfaction. The finding that the *Body Project* reduces caudate response to thin models provides novel preliminary evidence that this intervention reduces valuation of media images thought to contribute to body dissatisfaction and eating disorders, providing support for the intervention theory by documenting that this intervention alters an objective biological outcome.

## Introduction

Between 10–13% of women will experience an eating disorder, which are marked by greater distress, impairment, and morbidity than other psychiatric disorders, and increase risk for subsequent mood, anxiety and substance use disorders [1–4]. Unfortunately, 80% of adolescents and 57% of adults with eating disorders never receive treatment [4, 5] and lasting remission occurs in 50% or less of treated individuals [6–8]. Thus, a public health priority is to develop and disseminate effective eating disorder prevention programs.

Considerable support has emerged for a dissonance-based prevention program (the *Body Project*) that is based on the counter-attitudinal advocacy paradigm studied extensively in the field of persuasion [9]. In this paradigm, participants are induced to publically advocate for a topic inconsistent with their initial attitude, which theoretically generates cognitive dissonance that motivates participants to reduce their subscription to their original attitude. This is thought to occur because humans prefer to maintain consistency between behaviors and attitudes [10]. The *Body Project* targets women with body dissatisfaction because they are at high-risk for future eating disorders [11, 12]. In contrast with their typical beliefs, participants are induced to critique the thin ideal in verbal, written, and behavioral exercises (e.g., write a letter about the costs of pursuing this ideal). Publically criticizing the thin ideal theoretically prompts participants to reduce their subscription to this ideal, to maintain consistency between their behaviors and attitudes, which putatively decreases body dissatisfaction, negative affect, unhealthy weight control behaviors, eating disorder symptoms, and future eating disorder onset. The *Body Project* has produced greater reductions in eating disorder risk factors (e.g., thin-ideal internalization, body dissatisfaction), eating disorder symptoms, and eating disorder onset over 3-year follow-up than assessment-only control conditions and alternative prevention programs in randomized efficacy and effectiveness trails conducted by our research team [3, 13–16] and independent teams [16–19]. The *Body Project* is one of only two prevention programs that has reduced eating disorder onset, produced effects through 3-year follow-ups, and outperformed alternative interventions, and is the only program to produce independently replicated effects.

Evidence supports the assertion that the *Body Project* alters thinking patterns that contribute to risk for eating disorder onset. Consistent with the intervention theory, reductions in thin-ideal internalization and body dissatisfaction mediate the *Body Project* effects on change in eating disorder symptoms [20, 21] and participants assigned to high- versus low-dissonance versions of this program showed greater eating disorder symptom reduction [22, 23]. It also produced larger symptom reductions for youth with initially elevated thin-ideal internalization, consistent with the thesis that they experience more dissonance [18] and eliminated the negative effect of exposure to thin models on body dissatisfaction in young girls [14]. Although results suggest that dissonance-based prevention alters cognitive processing biases thought to contribute to eating disorders, it would be useful to document that the *Body Project* alters an objective biological outcome because demand characteristics inherent to randomized trials may have contributed to the effects on self-reported outcomes noted previously.

Experiments assessing the mechanism of effect for another widely studied dissonance paradigm imply that functional magnetic resonance imaging (fMRI) might capture the mechanism of effect for the *Body Project*. Numerous post-decision paradigm experiments have found that when participants choose between two similarly valued alternatives they subsequently rate the selected option more favorably and the rejected option less favorably [24–26]. Theoretically, choosing between two equally valued alternatives prompts cognitive dissonance that leads people to increase valuation of the selected option and decrease valuation of the rejected option (i.e., people alter their attitudes to be consistent with their behavior). Critically, Sharot et al. [26] found greater fMRI-assessed caudate activation when imagining the selected versus rejected option, and that change in caudate activation correlated with change in desirability ratings of the options (*r* = .63). These data link blood oxygen level dependent (BOLD) signal to degree of valuation. Another post-decision experiment found that increases in desirability ratings correlated most strongly with increases in ventral striatum BOLD activation during the choice process [25]. Evidence suggests that the caudate plays a role in encoding the reward value of stimuli [27, 28], implying that behaviorally committing to an alternative increases the biological representation of the expected value of that alternative [26]. However, because the orbitofrontal cortex [29] and amygdala [30] have also been implicated in encoding reward valuation, we conducted whole-brain analyses.

The previously reviewed findings prompted us to hypothesize that criticizing the thin ideal in the *Body Project* would lead to a greater reduction in responsivity in reward valuation regions to images of thin versus average-weight female models compared to control participants. Thus, we randomized participants to the *Body Project* or a control condition in which they received educational brochures on body image disturbances and eating disorders (see [3] for greater details). Participants underwent pre and post intervention fMRI scans while evaluating images of thin and average-weight models. We also tested whether pre-post changes in reward valuation region response correlated with pre-post changes in thin-ideal internalization, body dissatisfaction, and eating disorder symptoms.

## Methods

### Characteristics of the sample

Participants were 38 female adolescents (*M* age = 19.8 ± 2.2; M BMI = 24.5 ± 5.2; 13% Latino, 8% Asian/Pacific Islander, 3% African Americans, 68% European Americans, 5% Native Americans, and 3% mixed racial heritage) who were recruited from the University of Oregon via mass email messages and posted fliers for a trial evaluating a body acceptance intervention. Participants for the present study were recruited after we closed enrollment for a larger multi-site effectiveness trial of the *Body Project* among college students [3]. A brief phone screen interview that included items from the Schedule for Affective Disorders and Schizophrenia for School Age Children—Epidemiologic Version 5 (K-SADS-E 5; [31]) was used to verify inclusion and exclusion criteria. Inclusion criteria were self-reported body image concerns and female gender. Exclusion criteria were current use of psychoactive medications or drugs more than weekly, pregnancy, head injury with a loss of consciousness, significant cognitive impairment, major medical problems (e.g., diabetes), or current Axis I psychiatric disorder (including eating disorders).

The Oregon Research Institute Institutional Review Board (IRB) approved this study. Participants provided their written, informed consent (all had attained the legal age for consent) to participate, using an IRB-approved procedure. Participants completed surveys, interviews, and fMRI scans at pretest and 4 weeks later at posttest.

### Body Project Eating Disorder Prevention Program

The *Body Project* is a dissonance-based eating disorder prevention program delivered to groups of 6–10 participants by one to two clinicians. In session 1, participants collectively define the thin ideal promoted in Western culture and discuss costs of pursuing this ideal through a series of Socratic questions posed by facilitators, and are assigned home exercises (write an essay about the costs associated with pursuing the thin ideal; stand in front of a mirror with minimal clothing and record positive attributes about their bodies). In session 2, participants discuss the two home exercises, dissuade facilitators from pursuing the thin ideal in role-plays, and are assigned more exercises (write a letter to someone who pressured the participant to be thin, discussing the adverse effects; generate a top-10 list of things young women can do to challenge the thin ideal). In session 3, participants discuss the two home exercises, conduct role-plays challenging thin-ideal statements, discuss personal body image concerns, and are assigned home exercises (engage in a behavior that challenges their body image concerns; engage in two activities that challenge the thin ideal; write a letter to a younger self about how to avoid body image concerns). In session 4, participants discuss the three home exercises, discuss perceived benefits of the group intervention, and are assigned exit home exercises (commit to doing a self-affirmation activity that will promote body acceptance, encourage other young women at their school to complete the *Body Project* intervention, participant in some type of body activism activity as a group). This intervention was designed to underscore the voluntary nature of participation in the intervention, maximize accountability for the positions argued by participants, and maximize the level of effort, as these three factors optimize dissonance-induction. To underscore the voluntary nature of the intervention, participants were (a) reminded that participation was voluntary at the start of each session and (b) told that homework was not required. To increase accountability (a) sessions are video-recorded, (b) participants print and sign their name on each homework form, which are collected by the facilitator, (c) participants are encouraged to post their home exercises on a *Body Project* Internet page, and (d) participants are not told that topics discussed in sessions were confidential. To increase the level of effort (a) all homework assignments were made relatively difficult and (b) a high level of verbal participation was encouraged in sessions for each participant.

### Questionnaires and Interviews

The Ideal-Body Stereotype Scale-Revised assessed thin-ideal internalization; it has shown internal consistency (α = .91), 2-week test-retest reliability (*r* = .80), predictive validity for eating disorder symptom onset, and sensitivity to detecting eating disorder prevention program effects [18] (α = .78 at pretest). The Satisfaction and Dissatisfaction with Body Parts Scale [32] assessed dissatisfaction with nine body parts; it has shown internal consistency (α = .94), 3-week test-retest reliability (*r* = .90), predictive validity for eating disorder symptom onset, and sensitivity to detecting eating disorder prevention program effects [18] (α = .91 at pretest). The Eating Disorder Diagnostic Interview assessed DSM-IV eating disorder symptoms. Items assessing symptoms in the past month were summed to form an overall eating disorder symptom composite. This composite has shown internal consistency (α = .92), 1-week test-retest reliability (*r* = .90), inter-rater agreement (ICC *r* = .93), and sensitivity to detecting eating disorder prevention and treatment intervention effects [3] (α = .91 at pretest).

### Supervision, fidelity ratings, and competence ratings

Supervisors reviewed videotapes of the facilitator’s first group and a randomly selected 50% of the second group. Groups contained 8 participants each. The facilitator was sent supervisory e-mail messages praising positive behaviors and offering constructive suggestions. Supervisors independently coded a randomly selected 50% of sessions for intervention fidelity and competence on scales ranging from 1 to 10, showing inter-rater agreement for fidelity (ICC = .65) and competence (ICC = .72). Greater methodological details are provided in Stice et al., [3].

### fMRI paradigm

**Fig 1:**
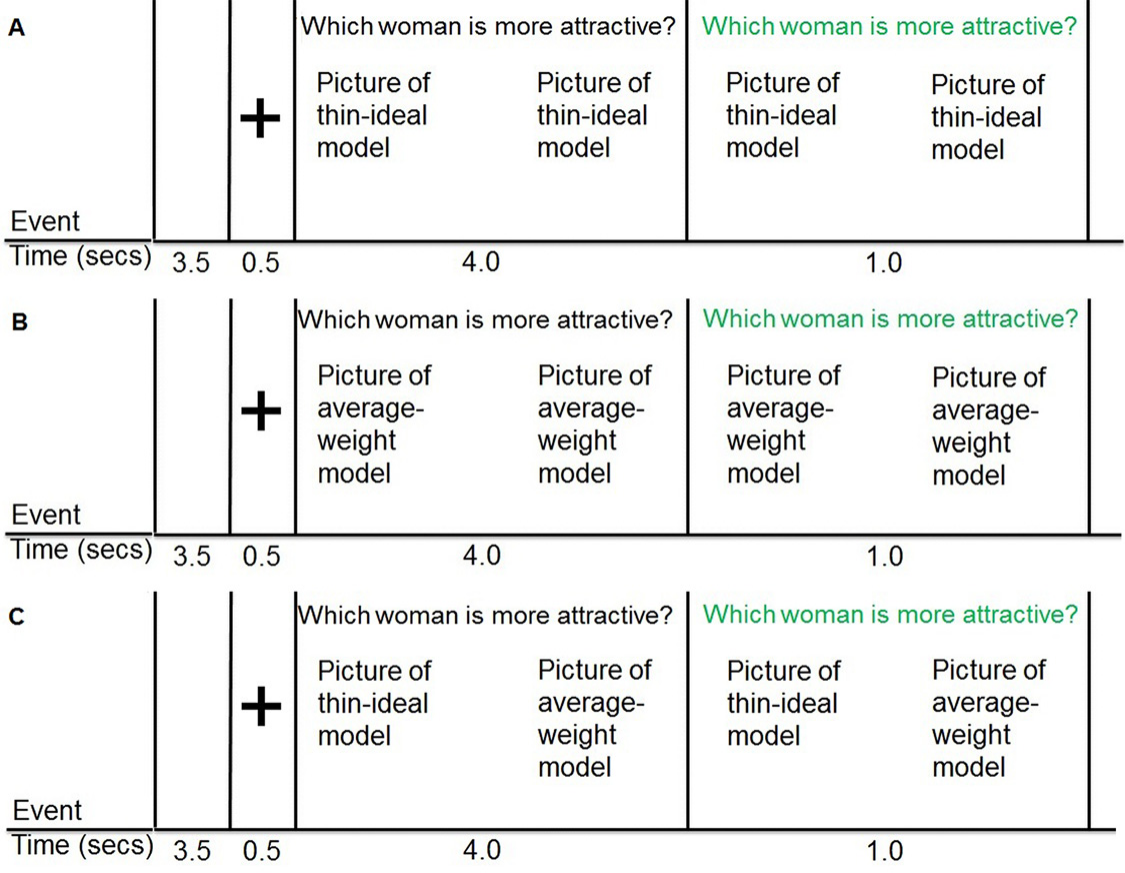
An illustration of the timing and ordering of our event-related thin image paradigm with the presentation of a) two thin-ideal models, b) two average-weight models, and c) one thin-ideal model and one average-weight model.

The event-related thin model picture paradigm (Fig 1) was designed to examine BOLD activation in response to 2 female models presented next to each other on the screen. Stimuli were presented in one scanning run (13 minutes) with a total of 60 events: 20 sets with two thin models, 20 sets with two average-weight models, and 20 sets with one thin model and one average-weight model. We included this latter image set (i.e., one thin and one average-weight model) to provide a behavioral test of whether *Body Project* participants show a greater decrease in the frequency of rating the thin model as more attractive than the average-weight model relative to changes observed in controls. A blank screen (duration 3.5 secs) was presented, followed by a blank screen with a cross-hair at the center (duration 0.5 secs) to prepare the subjects for the upcoming image set. Two pictures were presented next to each other with text in black font appearing above the images (“which woman is more attractive?”). We included this rating to prompt a focus on the appearance of the models. After 4 secs, the font of the text turned green (duration 1 sec) and participants were asked to respond by indicating their choice on a button box. Order of presentation of the picture pairs and the location (left or right) of the pictures were randomized across participants. To prevent order and location habituation from pre- to post, order of picture pairs and location of the images were also randomized within subjects.

### fMRI data acquisition, preprocessing, and analysis

Scanning was performed on a Siemens Allegra 3 Tesla head-only MRI scanner. Each scanning session started with a scout image to determine the position of the brain in the magnet. Pertinent image slice positions were based on the scout image. Functional scans used a T2*- weighted gradient single-shot echo planar imaging (EPI) sequence (echo time = 30ms, repetition time = 2000ms, flip angle = 80°) with an in plane resolution of 3.0 x 3.0 mm^2^ (64 x 64 matrix; 192 x 192 mm^2^ field of view). To cover the whole brain, 32 4mm slices (interleaved acquisition, no skip) were acquired along the AC-PC transverse, oblique plane as determined by the midsagittal section. A high resolution, T1- weighted 3D volume was acquired (MP-Rage with a TR/TE of 2100ms/2.4ms, flip angle of 15°, TI of 1100ms, matrix size of 256x256, FOV of 22cm, slice thickness of 1mm). Rather than include motion regressors in analyses, a prospective acquisition correction (PACE) was used to adjust slice position and orientation, as well as to re-grid residual volume-to-volume motion in real-time during data acquisition for the purpose of reducing motion-induced effects [33].

Data were pre-processed and analyzed using SPM8 (Wellcome Department of Imaging Neuroscience, London, England) in MATLAB (Mathworks, Inc., Sherborn, MA). Images were manually reoriented to the AC-PC line and skull stripped. Functional images were realigned to the mean and the anatomical and functional images were normalized to the standard Montreal Neurological Institute (MNI) T1 template brain (ICBM152). Normalization resulted in a voxel size of 3 mm^3^ for functional images and 1 mm^3^ for high-resolution anatomical images. Functional images were smoothed with a 6 mm full width at half maximum (FWHM) isotropic Gaussian kernel. Data were visually inspected for spikes, global mean signal fluctuations, ghosting artefacts, and subject motion within volume acquisition. No participant’s data set failed to meet the movement inclusion criteria, which were that within-run movement before correction did not exceed 2 mm in translational movement and 2° in rotational movement. For smaller movements, PACE adjusts slice position, orientation and regrids the residual volume-to-volume motion during data acquisition.

To identify brain regions activated in response to thin models we contrasted BOLD activation during the 4 sec presentation (before respond cue) of two thin models versus the 4 sec presentation of two average-weight models (i.e., images with 2 thin models > images with 2 average-weight models). Condition-specific effects at each voxel were estimated using general linear models. Vectors of the onset for each event of interest were compiled and entered into the design matrix so that event-related responses could be modeled by the canonical hemodynamic response function, as implemented in SPM8. A 128 second high-pass filter removed low-frequency noise and signal drift.

Individual maps were constructed to compare the activations within each participant for the aforementioned contrast and were constructed for pretest and posttest separately (i.e., pretest 2 thin models > pretest 2 average-weight models; posttest 2 thin models > posttest 2 average-weight models). We then conducted a 2 Group (intervention, control) x 2 Time (pre, post) repeated-measures ANOVA on BOLD responses to examine group differences in change in neural response to thin models between the *Body Project* and control group using these individual maps. Second, separate regression models tested whether whole-brain assessed pre-post changes in BOLD response correlated with pre-post changes in thin-ideal internalization, body dissatisfaction, and eating disorder symptoms. For these latter analyses, we created individual maps assessing the change in neural response between the 2 scan days: ([posttest 2 thin models > posttest 2 average-weight models] minus [pretest 2 thin models > pretest 2 average-weight models]) and correlated activity in these maps with change in thin-ideal internalization, body dissatisfaction, and eating disorder symptoms. Eating disorder symptoms were normalized with a square root transformation.

A gray matter mask of the whole brain was derived from the sample using Diffeomorphic Anatomical Registration Through Exponentiated Lie Algebra (DARTEL) segmentation in SPM following standard methods in Voxel-Based Morphometry (VBM8) [34]. Once segmented, the mean gray matter was resliced to 3 mm^3^ and binarized using the image calculator function in SPM at the level of i1>.03. Whole brain analyses were conducted after the binarized DARTEL derived sample-specific gray matter mask was applied. An overall significance level of p < 0.05 corrected for multiple comparisons across the gray matter masked whole brain was calculated. This calculation was accomplished by: 1) estimating the inherent smoothness of the masked functional data with the 3dFWHMx module in AFNI [35]; and 2) performing 10,000 Monte Carlo simulations of random noise at 3 mm^3^ through the masked data using the 3DClustSim module of AFNI [36]. This resulted in an inherent smoothness of 7.6322 and a threshold of *p <* 0.005 with cluster *k* ≥33 (two-sided test). This threshold has an overall significance level of *p <* 0.05, corrected for multiple comparisons across the whole brain. This study was powered to detect differential change in BOLD response across conditions rather than change in self-report measures, which requires larger cell sizes. This prompted us to focus on the effect sizes rather than the inferential tests for the self-report measures. fMRI effect sizes (*r*) were derived from the Z-values (Z/√N). Data were inspected to ensure that influential outliers did not drive significant effects.

## Results

### Preliminary analyses

**Fig 2:**
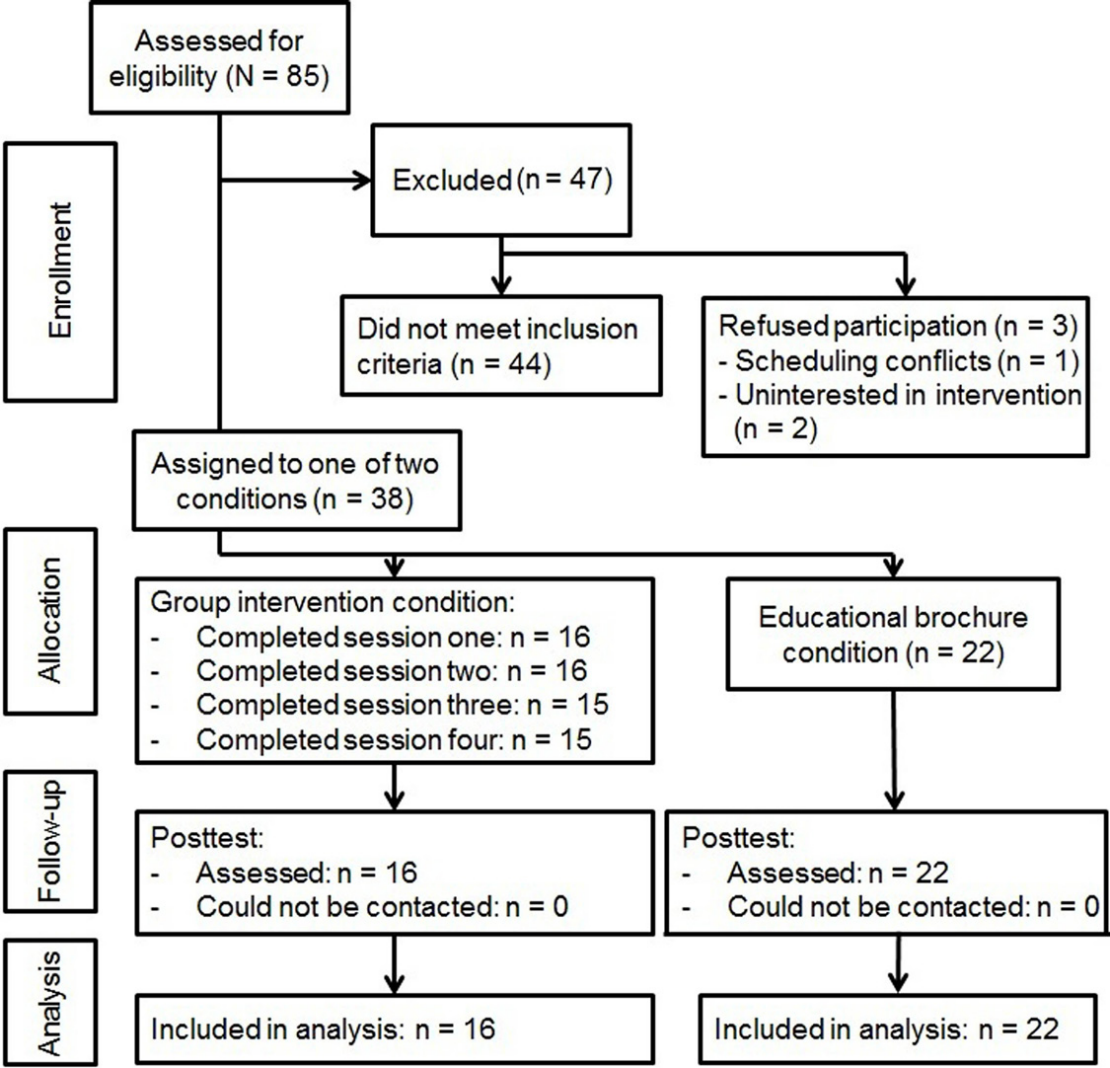
Participant Flow Chart.

Participants in the intervention and control conditions did not differ significantly on age, ethnicity, socioeconomic status, or pretest values of the outcome measures; Table 1 provides means and SD for outcomes at pre and post for each group. There was no attrition at posttest (see Fig 2 for the participant flow chart). Participants in the *Body Project* group condition either attended or made-up an average of 3.9 sessions (*SD* = 0.77); 94% attended or made-up all 4 sessions and 0% attended or made-up less than 2 sessions. Among those who missed a session, 89% received an individual make-up session. Participants completed 90% of the assigned home exercises. The mean fidelity rating was 7.8 (*SD* = 1.21) and the mean competence rating was 8.0 (*SD* = 1.13) on 1–10 point scales, suggesting that on average all key activities of each session were presented and with high therapist competence.

**Table 1:** Means (SD) for Outcomes in the Body Project and Control Conditions.

	Pretest		Posttest	
	*Mean*	*SD*	*Mean*	*SD*
Thin-ideal internalization				
*Body Project*	3.85	.35	3.54	.67
*Control group*	3.84	.37	3.73	.40
Body dissatisfaction				
*Body Project*	3.58	.54	2.96	.80
*Control group*	3.68	.55	3.35	.65
Eating disorder symptoms*				
*Body Project*	3.08	1.38	2.53	1.27
*Control group*	2.71	.81	2.47	.85

* square root transformation

There were no significant differences between the intervention group and control group in the mean amount of residual head movement across x, y, z rotation and translation (baseline translation *t*(36) = .35, *p* = .73; baseline rotation *t*(36) = .33, *p* = .74; post translation *t*(36) = -.67, *p* = .51; post rotation *t*(36) = .24, *p* = .82. Within-group effects on BOLD activity in response to thin versus average-weight models at pretest and posttest are presented in S1 and S2 Figs; S1 Table and S2 Table.

### Group differences in change in attractiveness ratings of thin versus average-weight models

*Body Project* (pretest *M* = 13.0 ± 4.3; posttest *M* = 10.6 ± 5.2) compared to the control participants (pretest *M* = 12.0 ± 5.2; posttest *M* = 11.9 ± 5.6) showed significant greater pre-post reductions in the number of times that they rated the thin model as more attractive than the average-weight model during the events when they had to choose between one thin and one average-weight model: (F_(1,36)_ = 4.40, *p* = 0.04, *d* = 0.70).

### Group differences in change in neural response to thin-ideal images

**Fig 3:**
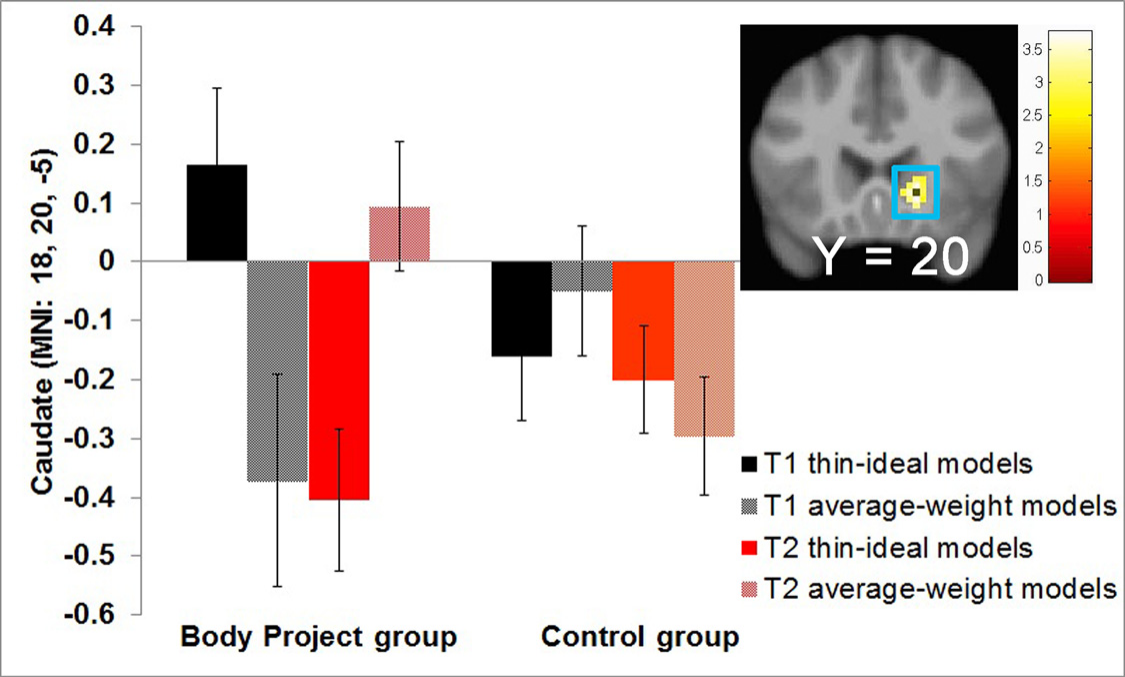
Greater pre to post BOLD response decreases in the right caudate (MNI: 18, 20, 5, Z = 3.66, *k* = 37; *r* = -0.59) in response to thin-models relative to average-weight models.

Whole brain analyses tested whether *Body Project* participants showed greater pre-post reductions in BOLD signal in response to viewing images of thin versus average-weight models compared to changes observed in controls. As hypothesized, *Body Project* participants showed a significantly greater decrease in BOLD activity in the right caudate (Fig 3; Table 2) compared to the control group. The two caudate peaks were the only ones to reach significance in the whole brain analyses. No significant effects emerged for the reversed contrasts. Because the caudate responses at pretest differed between the intervention group and the control group (Fig 3), it is possible that this interaction is partially driven by differences in BOLD activity before the intervention. We therefore tested for group differences in BOLD activity in response to viewing images of 2 thin versus 2 average-weight models at pretest by means of whole brain analyses. Although the intervention group showed significantly greater activation in several regions (S3 Table), there were no significant group differences in BOLD activity in the caudate. We then performed region-of-interest searches using the two peaks in the caudate (Table 2) as centroids to define 10-mm diameter spheres. Again, there were no significant group differences in BOLD activity in the caudate.

**Table 2:** Significant Time-by-Group Interactions in Brain Activation during Thin-Ideal Models vs Average-Weight Models.

Contrasts and regions	*k*	Z-value	MNI coordinates	r
***Thin-ideal models > average-weight models***				
Posttest>pretest: Body Project>control				
Caudate	37	3.66	18, 20, -5	-0.59
Caudate		3.35	9, 14, -5	-0.54

For all contrasts, activated regions, *Z*-values, and coordinates within the MNI coordinate system are displayed. Number of contiguous voxels (*k*) are shown for peak coordinates. Clusters may contain more than one brain region as indicated by multiple regions under one cluster size.

### Correlations of pre-post changes in BOLD signal with pre-post changes in thin-ideal internalization, body dissatisfaction, and eating disorder symptoms

The pre-post reductions in thin ideal internalization (F _(1,36)_ = 1.81, *p* = 0.19, *d* = 0.45), body dissatisfaction (F_(1,36)_ = 3.02, *p* = 0.09; *d* = 0.58), and eating disorder symptoms (F_(1,36)_ = 1.36, *p* = 0.25, *d* = 0.39) were not significantly larger for *Body Project* versus control participants in this study that was powered to detect changes in BOLD signal. However, the effects sizes were only somewhat smaller than those observed in the larger multi-site effectiveness trial that was conducted with this population immediately before participants were recruited for this fMRI study (corresponding *d*’s = .77, .64, & .54 respectively) [3].

**Fig 4:**
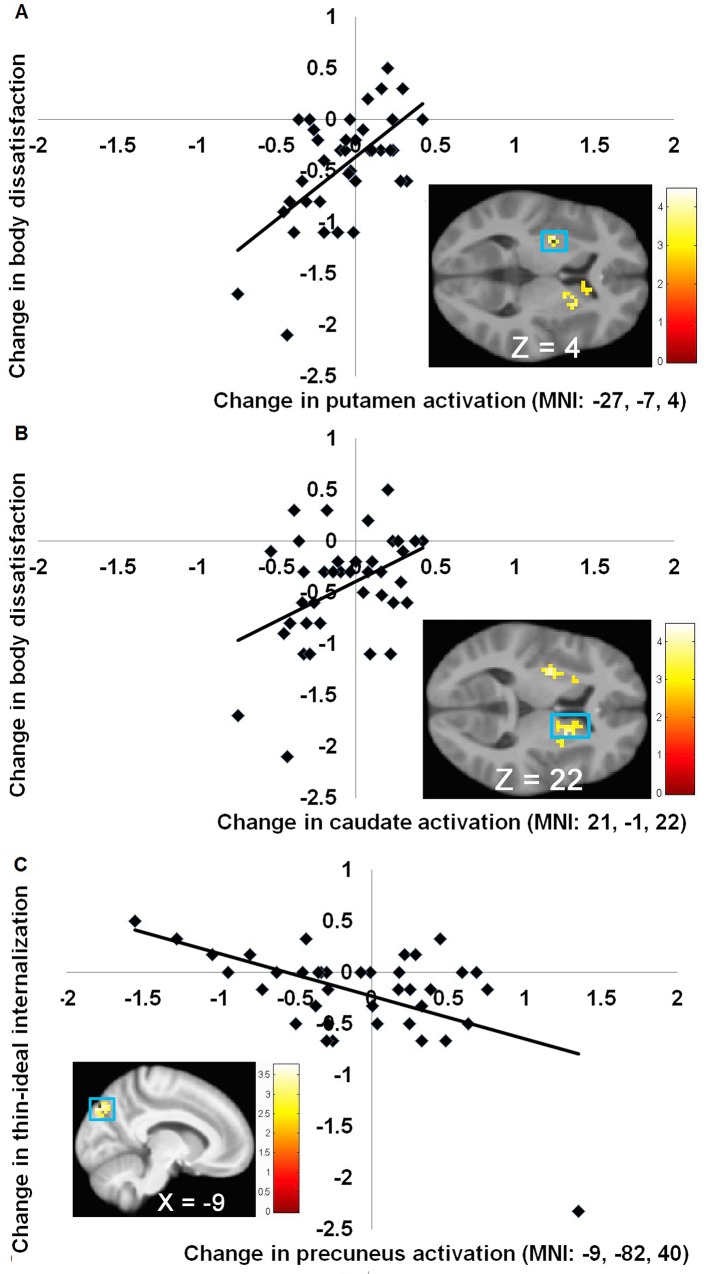
Scatter plots of relative activations. (**A**) The positive correlation between change in putamen activation and change in body dissatisfaction (*r* = 0.64). (**B**) The positive correlation between change in caudate activation and change in body dissatisfaction (*r* = 0. 63). (**C**) The negative correlation between change in precuneus activation and change in thin-ideal internalization (*r* = -0.55).

Whole brain analyses tested whether pre-post changes in BOLD signal in response to thin versus average-weight models correlated with pre-post changes in thin-ideal internalization, body dissatisfaction, and eating disorder symptoms. There were statistically significant, large positive correlations between reductions in bilateral putamen and caudate activation (Table 3), the two key regions that make up the dorsal striatum, and reductions in body dissatisfaction (*M r* = .60; Fig 4A and 4B). Further, greater pre-post increases in precuneus activation correlated with greater pre-post reductions in thin-ideal internalization (Fig 4C; Table 3). However, this peak became non-significant when excluding the case that showed the greatest decrease in thin-ideal internalization, suggesting that this peak was strongly driven by this outlier. There were no significant correlations between changes in BOLD signal in response to thin versus average-weight models and changes in eating disorder symptoms.

**Table 3:** Correlations between Changes in Brain Responsivity to Thin-Ideal Models versus Average-Weight Models and Changes in Body Dissatisfaction, Thin-Ideal Internalization, and Eating Disorder Symptoms (N = 38).

Contrasts and regions	*k*	Z-value	MNI coordinates	*r*	*p*-value
***(posttest Thin-ideal models > posttest average-weight models) > (pretest Thin-ideal models > pretest average-weight models)***					
Change in body dissatisfaction					
Putamen	78	3.96	-27, -7, 4	0.64	< .001
Caudate		3.48	-15, 8, 16	0.56	< .001
Caudate	138	3.88	21, -1, 22	0.63	< .001
Putamen		3.67	21, 5, 7	0.60	< .001
Caudate		3.43	15, 17, -5	0.56	.001
Change in thin-ideal internalization					
Precuneus	43	3.42	-9, -82, 40	-0.55	.001

For all contrasts, activated regions, *Z*-values, and coordinates within the MNI coordinate system are displayed. Number of contiguous voxels (*k*) are shown for peak coordinates.

## Discussion

As hypothesized, participants who completed the *Body Project*, a dissonance-based prevention program, showed significantly greater reductions in caudate responsivity to images of thin models than controls, which is important because these images are associated with body dissatisfaction and eating disorders. Interestingly, *Body Project* participants also showed a pre-post increase in caudate responsivity to average-weight models. We expected reductions in caudate response to thin models based on post-decision dissonance experiments that reported that caudate response to selected options increased and caudate response to rejected options decreased after making a choice between two equally desired alternatives [26] and that post-decision increases in desirability ratings correlated with increased activation in the ventral striatum during the choice process [25]. It should be noted that we used whole-brain analyses and a conservative multiple testing correction, in contrast to the analytic approach used Sharot and associates [26]. As such, our findings from a trial involving the counter-attitudinal advocacy paradigm extend results from studies investigating the post-decision dissonance-induction paradigm. Further, we investigated the effects of a complex multifaceted behavioral intervention, versus a simple choice task. Given that the caudate has been implicated in tracking the reward valuation of stimuli [27, 28] and because women diagnosed with anorexia nervosa compared to healthy comparison women show greater caudate activation in response to underweight female images [37,38], results imply that the *Body Project* reduces valuation of thin models. This interpretation is also consistent with evidence that *Body Project* participants showed a significant pre-post reduction in the frequency of rating the thin model as more attractive than the average-weight model compared to controls, as well as a reduction in thin-ideal internalization. These results are encouraging because positive appraisal of thin models and pursuit of the thin ideal putatively contributes to body dissatisfaction and eating disorders [39,40].

Evidence that the *Body Project* reduces valuation of thin models also extends findings from the response training literature. In this training, computerized stop-signal, go/no-go, and respond-signal paradigms cue participants to withhold a behavioral response to some stimuli and to make a behavioral response to other stimuli. Response training also appears to influence valuation. For example, food images repeatedly paired with inhibition signals were subsequently rated as less palatable and valuable relative to foods repeatedly paired with response signals [41–43]. Also, elevated responsivity in regions implicated in valuation (ventral/mediodorsal striatal regions and ventral medial prefrontal cortex) to foods paired with response signals correlated with how often these foods were chosen [41]. Collectively, these data suggest that humans automatically devalue stimuli associated with behavioral inhibition and value stimuli associated with behavioral approach. It is possible that metaphorically “pushing the thin ideal away” in *Body Project* exercises serves to devalue the appearance ideal. This raises a provocative question regarding the degree to which cognitive dissonance processes (the desire for consistency between behaviors and attitudes) versus response training processes (pushing the thin ideal away in behavioral, verbal, and written activities) drive the effects of the *Body Project*. In some respects, the *Body Project* may provide a more potent and complex response training than the simple computer training paradigms that focus solely motor inhibition. Interestingly, there is evidence that behavioral choice induces valuation changes via relatively automatic processes that do not depend on explicit memory or complex cognitions [26]. For example, post-decision change in valuation has been found in amnesic patients with no explicit memory of choices [44]. Post-decision valuation is also retained in situations wherein complex cognition is decreased, such as when distractors are used to increase cognitive load, and when testing young children or monkeys [44,45]. One clinical implication is that research should test whether adding response inhibition training for thin models increases the intervention effects from the *Body Project*.

Pre-post reductions in caudate response to thin models correlated positively with pre-post reductions in body dissatisfaction, but not thin-ideal internalization or eating disorder symptoms. This suggests that valuation of thin models may play a more pronounced role in promoting body dissatisfaction than in reported subscription to the thin ideal or engaging in disordered eating. These results also seem consistent with the suggestion that body dissatisfaction is a key mediator of the effects of the *Body Project* [20]. Although change in thin-ideal internalization correlated with responsivity to thin versus average-weight models in the precuneus, this effect was driven by an outlier.

There are important limitations to consider when interpreting the results. First, although the sample was relatively large for an fMRI study, it was relatively small for behavioral self-report measures, which reduced sensitivity for the analyses involving the latter outcomes. However, it is reassuring that the intervention effect sizes were only slightly smaller than those observed in the larger trial from which participants were selected [3]. Second, because the fMRI paradigm included a fixed inter-trial interval rather than a variable inter-trial interval, the statistical efficiency of our design was not optimal and may have resulted in less accuracy in the estimation of the event-related hemodynamic response to the different stimuli. Future research might benefit from incorporating variable inter-trial intervals between the images. Third, despite the non-significant group differences in caudate activity at pretest, it is still possible that our central finding is partially driven by pretest group differences in neural activity in other brain regions functionally connected with the caudate. Independent replication is needed before definitive conclusions can be made about the effects of the Body Project on neural changes in reward valuation regions to images of thin models. Finally, it would have been optimal to repeat the fMRI scans after a several-month follow-up to confirm that the neural responsivity effects persist. Although the fact that effects of the *Body Project* have persisted through 3-year follow-up [18, 19] implies that intervention effects persist, future studies should investigate the persistence of valuation changes.

This experiment provides novel evidence that a dissonance-based prevention program reduces reward region response to thin models, which are thought to play a key role in the development of body dissatisfaction and eating disorders. Our findings suggest that this brief prevention program changes caudate responsivity when young women view media portrayals of the thin ideal. Striatal responses also correlated strongly with reductions in body dissatisfaction, a finding consistent with the theory that valuation of thin images plays a key role in body image concerns. Results are also important because they provide evidence that this prevention program alters an objective biological outcome, implying that the effects of this intervention are not simply due to demand characteristics inherent to randomized trials. Thus, these findings add to an accumulating evidence-base that support the intervention theory for this prevention program, which is a vital element of prevention science that should increase the likelihood of adoption and implementation of this prevention program.

## Supporting Information

S1 Dataset (SPSS). (SAV)

S1 Table Within-group comparisons for the intervention group (n = 16) at pretest and posttest contrasting 2 thin-ideal models versus 2 average-weight models in the thin-ideal paradigm. (DOCX)

S2 Table Within-group comparisons for the control group (n = 22) at pretest and posttest contrasting 2 thin-ideal models versus 2 average-weight models in the thin-ideal paradigm. (DOCX)

S3 Table Between-group comparisons at pretest in the thin-ideal paradigm. (DOCX)

S1 Fig Cortical activations in the intervention group (n = 16) plotting voxels more active during viewing two thin-ideal models compared to viewing two average-weight models at A) pretest and B) posttest. (DOCX)

S2 Fig Cortical activations in the control group (n = 22) plotting voxels more active during viewing two thin-ideal models compared to viewing two average-weight models at A) pretest and B) posttest. (DOCX)
