# Supplementary material for: Dissonance-Based Eating Disorder Prevention Program Reduces Reward Region Response to Thin Models; How Actions Shape Valuation
Source: PLoS One. 2015 Dec 7;10(12):e0144530. doi: 10.1371/journal.pone.0144530 (PMC4671712; doi:10.1371/journal.pone.0144530)
Supplement: S1 Table — (DOCX) [file pone.0144530.s002.docx]

S1 Table

Within-group comparisons for the intervention group (n = 16) at pretest and posttest contrasting 2 thin-ideal models versus 2 average-weight models in the thin-ideal paradigm

| Contrast and region | *k* | Z value | MNI coordinates |
| --- | --- | --- | --- |
| *Pretest thin-ideal models > pretest average-weight models* |  |  |  |
| Middle temporal gyrus | 325 | 5.12 | -45, -58, -5 |
| Inferior occipital gyrus |  | 5.01 | -51, -70, -8 |
| Inferior occipital gyrus |  | 4.49 | -48, -61, -14 |
| Middle occipital gyrus | 75 | 4.51 | -30, -85, 25 |
| Superior occipital gyrus |  | 4.05 | -15, -85, 40 |
| Inferior temporal gyrus | 212 | 4.32 | 45, -55, -8 |
| Inferior temporal gyrus |  | 3.91 | 48, -70, -8 |
| Inferior temporal gyrus |  | 3.21 | 54, -58, -14 |
| Superior parietal lobe | 423 | 4.19 | 24, -67, 61 |
| Superior parietal lobe |  | 3.88 | 27, -55, 67 |
| Inferior parietal lobe |  | 3.81 | 36, -40, 52 |
| Superior parietal lobe | 109 | 4.16 | -21, -67, 52 |
| Superior parietal lobe |  | 3.02 | -33, -52, 55 |
| Inferior parietal lobe | 107 | 4.05 | -63, -34, 31 |
| Inferior parietal lobe |  | 3.78 | -48, -40, 37 |
| Inferior parietal lobe |  | 3.40 | -54, -37, 49 |
| Cerebellum | 232 | 3.74 | 33, -37, -29 |
| Fusiform gyrus |  | 3.56 | 36, -73, -20 |
| Superior occipital gyrus | 76 | 3.68 | 27, -85, 28 |
| Superior occipital gyrus |  | 3.36 | 27, -79, 37 |
| Cerebellum | 97 | 3.52 | -18, -67, -26 |
| Fusiform gyrus |  | 3.48 | -27, -67, -17 |
| Precentral gyrus | 75 | 3.46 | 60, -1, 28 |
| Precentral gyrus |  | 3.26 | 51, -7, 37 |
| *Posttest thin-ideal models > posttest average-weight models* |  |  |  |
| Fusiform gyrus | 147 | 4.55 | -24, -70, -14 |
| Cerebellum |  | 3.37 | -9, -73, -20 |
| Cerebellum |  | 3.36 | -30, -67, -26 |
| Inferior temporal gyrus | 103 | 4.28 | -57, -64, -11 |
| Inferior occipital gyrus |  | 3.90 | -42, -67, -5 |
| Fusiform gyrus |  | 3.69 | -48, -61, -20 |
| Fusiform gyrus | 129 | 3.81 | 27, -64, -17 |
| Inferior occipital gyrus |  | 3.34 | -45, -76, -11 |

For all contrasts, activated regions, Brodmann areas (BA), *Z*-values, and coordinates within the MNI coordinate system are displayed. Number of contiguous voxels (*k*) are shown for peak coordinates. Clusters may contain more than one brain region as indicated by multiple names under one cluster size. Thresholds were determined with Monte Carlo simulations of random noise distribution using the AlphaSim module of AFNI. For analyses of the main effects no mask was applied. Peaks within the regions were considered significant at p<0.005, *k* ≥52, *p* < 0.05, corrected for multiple comparisons across the entire brain.
