# Supplementary material for: Dissonance-Based Eating Disorder Prevention Program Reduces Reward Region Response to Thin Models; How Actions Shape Valuation
Source: PLoS One. 2015 Dec 7;10(12):e0144530. doi: 10.1371/journal.pone.0144530 (PMC4671712; doi:10.1371/journal.pone.0144530)
Supplement: S2 Table — (DOCX) [file pone.0144530.s003.docx]

S2 Table

Within-group comparisons for the control group (n = 22) at pretest and posttest contrasting 2 thin-ideal models versus 2 average-weight models in the thin-ideal paradigm

| Contrast and region | *k* | Z value | MNI coordinates |
| --- | --- | --- | --- |
| *Pretest thin-ideal models > pretest average-weight models* |  |  |  |
| Inferior temporal gyrus | 1762 | 5.20 | 51, -64, -8 |
| Superior occipital gyrus |  | 4.64 | 15, -91, 22 |
| Inferior parietal lobe |  | 4.64 | 63, -34, 28 |
| Inferior parietal lobe | 1068 | 4.19 | -57, -40, 25 |
| Middle occipital gyrus |  | 4.15 | -36, -76, -2 |
| Middle temporal gyrus |  | 4.14 | -42, -79, 7 |
| Mid cingulate gyrus | 82 | 4.09 | 3, -7, 37 |
| Mid cingulate gyrus |  | 3.20 | 0, 5, 40 |
| Fusiform gyrus | 85 | 3.97 | -27, -64, -14 |
| Cerebellum |  | 3.17 | -27, -76, -20 |
| Cerebellum |  | 3.02 | -33, -61, -23 |
| Mid cingulate cortex | 97 | 3.74 | -6, -40, 52 |
| *Posttest thin-ideal models > posttest average-weight models* |  |  |  |
| Middle temporal gyrus | 2699 | 4.96 | 45, -58, -2 |
| Superior parietal lobe |  | 4.94 | 24, -61, 58 |
| Fusiform gyrus |  | 4.72 | 36, -52, -14 |
| Hippocampus | 81 | 4.20 | 21, -34, -5 |
| Hippocampus |  | 3.74 | 27, -28, -11 |

For all contrasts, activated regions, Brodmann areas (BA), *Z*-values, and coordinates within the MNI coordinate system are displayed. Number of contiguous voxels (*k*) are shown for peak coordinates. Clusters may contain more than one brain region as indicated by multiple names under one cluster size. Thresholds were determined with Monte Carlo simulations of random noise distribution using the AlphaSim module of AFNI. For analyses of the main effects no mask was applied. Peaks within the regions were considered significant at p<0.005, *k* ≥74, *p* < 0.05, corrected for multiple comparisons across the entire brain.
