# Supplementary material for: Dissonance-Based Eating Disorder Prevention Program Reduces Reward Region Response to Thin Models; How Actions Shape Valuation
Source: PLoS One. 2015 Dec 7;10(12):e0144530. doi: 10.1371/journal.pone.0144530 (PMC4671712; doi:10.1371/journal.pone.0144530)

S1 Figure

Cortical activations in the intervention group (n = 16) plotting voxels more active during viewing two thin-ideal models compared to viewing two average-weight models at **A**) pretest and **B**) posttest


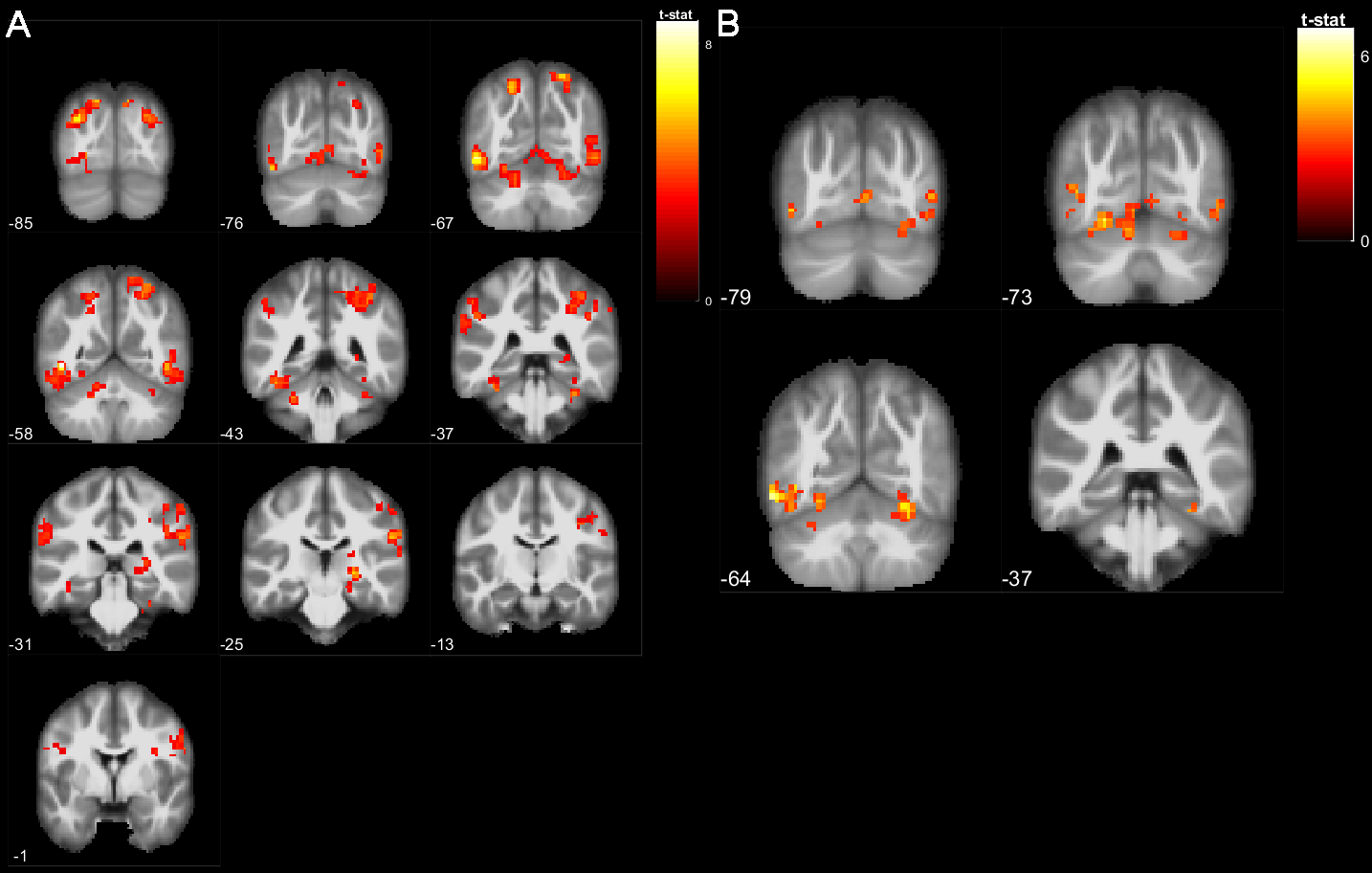

Supplement: S1 Fig — (DOCX) [file pone.0144530.s005.docx]
